# An Orientation Sensor-Based Head Tracking System for Driver Behaviour Monitoring

**DOI:** 10.3390/s17112692

**Published:** 2017-11-22

**Authors:** Yifan Zhao, Lorenz Görne, Iek-Man Yuen, Dongpu Cao, Mark Sullman, Daniel Auger, Chen Lv, Huaji Wang, Rebecca Matthias, Lee Skrypchuk, Alexandros Mouzakitis

**Affiliations:** 1 School of Aerospace, Transport and Manufacturing, Cranfield University, Cranfield MK43 0AL, UK; lorenz.goerne@lmtnet.de (L.G.); ewunmy@gmail.com (I.-M.Y.); d.cao@cranfield.ac.uk (D.C.); m.sullman@cranfield.ac.uk (M.S.); d.j.auger@cranfield.ac.uk (D.A.); C.Lyu@cranfield.ac.uk (C.L.); Huaji.Wang@cranfield.ac.uk (H.W.); 2 Jaguar Land Rover Limited, University Road, University of Warwick, Coventry CV4 7AL, UK; rmatthia@jaguarlandrover.com (R.M.); lskrypch@jaguarlandrover.com (L.S.); amouzak1@jaguarlandrover.com (A.M.)

**Keywords:** computer vision, non-driving activities, autonomous driving, attention level

## Abstract

Although at present legislation does not allow drivers in a Level 3 autonomous vehicle to engage in a secondary task, there may become a time when it does. Monitoring the behaviour of drivers engaging in various non-driving activities (NDAs) is crucial to decide how well the driver will be able to take over control of the vehicle. One limitation of the commonly used face-based head tracking system, using cameras, is that sufficient features of the face must be visible, which limits the detectable angle of head movement and thereby measurable NDAs, unless multiple cameras are used. This paper proposes a novel orientation sensor based head tracking system that includes twin devices, one of which measures the movement of the vehicle while the other measures the absolute movement of the head. Measurement error in the shaking and nodding axes were less than 0.4°, while error in the rolling axis was less than 2°. Comparison with a camera-based system, through in-house tests and on-road tests, showed that the main advantage of the proposed system is the ability to detect angles larger than 20° in the shaking and nodding axes. Finally, a case study demonstrated that the measurement of the shaking and nodding angles, produced from the proposed system, can effectively characterise the drivers’ behaviour while engaged in the NDAs of chatting to a passenger and playing on a smartphone.

## 1. Introduction

The autonomous vehicle is one of the next generation trends in vehicle development. According to SAE International Standard J3016 [1], Level 3 vehicle automation, or highly automated driving (HAD), presents an exciting new development in the field of driving research and technology. The European roadmap has suggested Level 3 automation will be available at low speed and in less complex driving scenarios by 2020. Higher level autonomy is expected to be available on motorways by 2025 and in cities by 2030. Although at present legislation does not allow drivers in a Level 3 autonomous vehicle to engage in non-driving activities (NDAs), HAD may in the future allow drivers to more freely engage in NDAs during much of the time while the automated system monitors and reacts to the driving environment. HAD is not entirely autonomous and at some points during a journey (for example when approaching a complex or less predictable driving scenario, such as temporary road works) the driver will be required to disengage from their NDA and return to the driving task. This suggests a new form of driver interaction with the vehicle and poses new challenges in the science of driving, namely how to achieve a pleasurable driving experience that allows for engagement in NDAs. However, the development of HAD also places a new demand on the driver, which will require the switch from NDA engagement to driving the vehicle, whenever requested by the system. Therefore, the task of driver monitoring will change from monitoring the drivers’ inattention level while driving to monitoring the drivers’ attention level while engaged in a NDA. The measurement of attention level will play an important role in determining when a driver can be given control of the vehicle. The main challenges here are that the potential NDAs are diverse and that drivers may engage in more complex NDAs than during standard driving. According to Sivak and Schoettle [2], the most common NDAs UK drivers reported wanting to engage in were: reading (7.6%), sleeping (7.2%), texting or talking with friends/family (5.5%), working (4.9%), watching movies/TV (4.2%), and playing games (1.9%). Developing a robust monitoring system to fully cover these NDAs is a substantial challenge.

Apart from eye movements [3], the tracking of which is particularly vulnerable to lighting conditions, head tracking is one of the most commonly used approaches for studying driver attention [4,5,6,7,8,9,10]. The connection between visual focus and head pose estimation has been studied to construct a distribution model [11]. Asteriadis et al. [12] and Mbouna et al. [13] presented their research on observers’ attention level by analysing both gaze direction and head pose estimation. Interestingly, recently head motion, along with lane position and vehicle dynamics, has been used to predict a driver’s intent to turn [14] and change lanes [15]. However, these studies have focused on drivers who were actively driving, and very little research has been conducted on drivers engaged in NDAs, based on head movement. Many state-of-the-art vision-based head pose algorithms have taken the necessary steps to be automatic, fast, and person invariant [16]. The most commonly used approach to measure the orientation of the head is reliant upon cameras and the associated image analysis algorithms. The techniques range from classic knowledge based 3D-models which are projected and fitted on the image, to modern algorithms based on machine learning and artificial neural networks [17]. One limitation of these approaches is that the accuracy dramatically reduces due to changes in light conditions, or any other disturbances. For example, the driver’s face may be occluded by cosmetic accessories (e.g., scarfs, hats, glasses, chains). Another disadvantage is that the performance is limited to within a certain range of angles. The range of head motion for an average adult male encompasses sagittal flexion and extension (i.e., forward to backward movement of the neck) from −60.4° to +69.6°, frontal lateral bending (i.e., right to left bending of the neck) from −40.9° to +36.3°, and horizontal axial rotation (i.e., right to left rotation of the head) from −79.8° to +75.3° [18]. The present research shows that a single camera can only cover from −20° to +20°, meaning that current approaches will fail if there are large head movements in any direction. A natural choice for the design of a more flexible system is through the use of multiple-cameras [19], but the cost of such a system and the computational time will also be increased.

This paper reports the development of a new head movement monitoring system which is based on twin orientation sensors in order to improve fidelity and the robustness of head tracking. This new system is then evaluated by studying a driver engaging in selected NDA tasks while traveling in a simulated Level 3 autonomous vehicle.

## 2. Materials and Methods

### 2.1. Orientation-Sensor-Based Head Tracking

Since more and more portable devices (e.g., smartphones) demand orientation data for many of their features, the availability and accuracy of these sensors has improved dramatically. Orientation sensors are now an inexpensive and easy-to-use method for 3D tracking. Due to their small size, they can easily be integrated into portable devices for monitoring head movements within a vehicle. The potential for orientation sensors to be used in a moving platform, like planes or vehicles, has been investigated based on static simulators [17]. A limited number of studies have also reported the use of small-scale inertia measurement units in a moving platform [20,21,22]. Solutions which use this approach to detect head movement, with an accuracy of <1° per axis, are commercially available [23], but they are costly and usually do not work on a moving platform. This paper proposes a solution which uses two independent sensors, one of which measures head movement and the other measures vehicle movement. Measuring vehicle movement is important, as it means that the effect of the vehicle movement on head movement can be removed.

#### 2.1.1. Sensors

**Figure 1:**
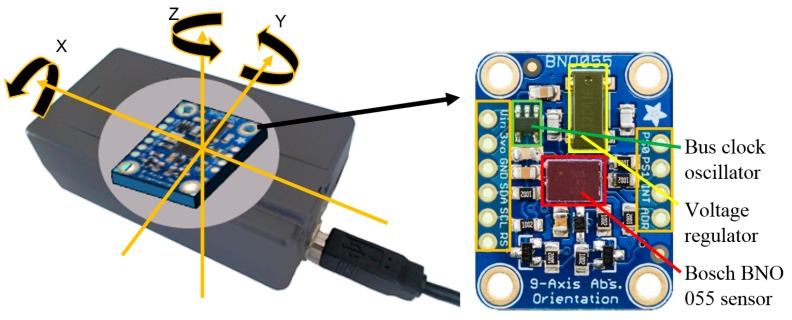
Designed head movement tracking device (left) and the BNO 055 on an Adafruit™ breakout board.

The sensor chosen for measuring head orientation was a BNO 055 (Bosch^®^, Reutlingen, Germany). This is a relatively new chip that is designed for high fidelity navigation applications in portable devices. It includes three triaxial sensors for measuring acceleration, rotation speed and magnetic fields, respectively. The three measures are fused by an internal logic chip that calculates the orientation of the system relative to a geostatic coordinate system that aligns with the earth’s magnetic and gravitational field. The logic chip also compensates for the effect of temperature on the sensors and automatically calibrates them. For the gyroscope, the calibration routine automatically removes any drift through resting the device in a static position for a few seconds. The accelerometer is calibrated by switching between 6 static positions, each of which is perpendicular to the other. The magnetometer initially calibrates itself by drawing the ‘8’ pattern in the air, and then dynamically compensates for the effect of variations in the magnetic field, while being moved. Such an approach generates reliable tracking over a long period and minimises the drift that occurs during measurement. The proposed device benefits from the high sampling resolution (16 bits) and an internal filter that effectively removes noise from the output. The data can be retrieved by communicating via the I2C protocol, which is a popular standard that is supported by many microprocessors. Due to the low-pass filtering process, the maximal sampling rate is 100 Hz. A snapshot of the device is shown in Figure 1.

#### 2.1.2. Sensor Fusion

On a static platform, a tri-axial accelerometer is sufficient to determine the nodding (*X*) and rolling (*Y*) axes. The earth’s gravitational field provides the necessary reference to determine the shaking (*Z*) axis. Since the proposed system will be used on a moving platform, this device uses a gyroscope which detects the rotation speed to compensate for disturbances, such as vibration or vehicle acceleration. Rotation speed is integrated to yield a secondary measurement for the rotation angle and both values are then fused to a single measurement using a Kalman filter. Another problem is that the system’s *Z* axis will drift over time due to inaccuracy in the sensor that accumulates over time. The BNO 055 uses a tri-axial magnetometer to provide another reference.

#### 2.1.3. Communication

**Figure 2:**
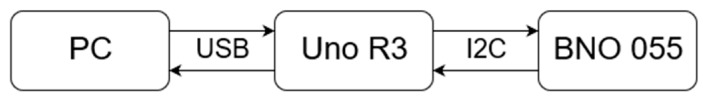
Data flow of the communication.

The Uno R3 (Arduino^®^, Turin, Italy) was chosen as the communication gateway between the device and PC. This is a popular platform in the low-price segment of the market that has many compatible parts. The variety of Input/Output-ports and the fact that the power input can be provided via a cable or battery make this platform a prototyping-friendly solution. The Uno R3 connects to a PC via the standard USB port, which enables the system to be used in conjunction with a variety of operating systems, such as Windows or Linux. A Windows serial port is used as the communication protocol. The set of commands that can be used to control the device are called by software that runs on the PC. The maximal sampling rate of this protocol is about 200 Hz, which is acceptable for this system as it is twice the maximal sampling rate of the sensor. The data flow is illustrated by Figure 2. 

#### 2.1.4. Attachment

It is important to ensure the sensor has a firm connection to the head, so that there is as little relative movement as possible. A generic head strap that is intended for action cameras was used. The strap has a support that goes over the head, which minimises movement in the Z direction, and can be adapted to each individual by changing the length of the straps. 

#### 2.1.5. Coordinate System

**Figure 3:**
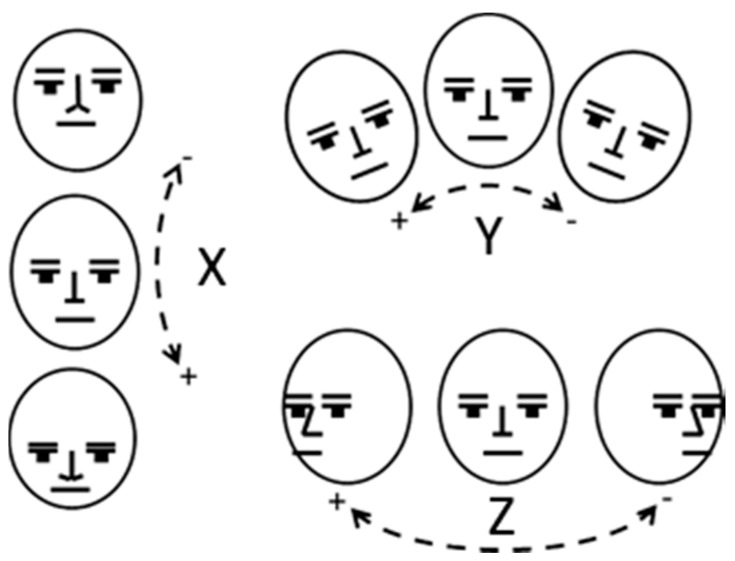
Head’s principle axes of motion [16], where X denotes pitch (nodding), Y denotes roll and Z denotes yaw (shaking).

This study also used a camera-based head tracking system to compare with the system developed here. Establishing the synchronisation between the two devices is important. Figure 3 illustrates the typical definition of head movement in 3 axes. The data produced by the head tracking device are relative to a geostatic reference frame. To ensure that the axes of the device align with the head, an initial calibration was required. This was accomplished through changing the position of the device by moving the elastic head strap. The testing person faced towards the camera and the device was moved until the orientation data of the device reached the origin point. If a different reference frame was required, an additional calibration routine was performed so that the head orientation data could be transformed to the reference frame. To do so, the individual being tested faced straight towards the camera and the Euler angles, which correspond to this position, were saved. This vector of angles was then subtracted from all future measurement, as expressed below:

(1) $$\vec{o_{cam}}=\vec{o_{measure}}-\vec{o_{calib}}$$

where $\vec{o_{cam}}$ is the set of Euler angles in the camera reference frame, $\vec{o_{measure}}$ are the current measured orientation Euler angles and $\vec{o_{calib}}$ are the Euler angles which are saved from the previous calibration process. 

Since the driver is on a moving platform, one device with a geostatic reference could not separate vehicle movement and head movement. To solve this problem, a second identical device was used to monitor the vehicle movement only. The device must be positioned so that its axes align with the vehicle’s main axes. This can be done using the same manner as for the participant being monitored. The final set of Euler angles, that describe the head’s orientation, are calculated by:

(2) $$Z=Z_{H}-Z_{V}$$

(3) $$Y=Y_{H}-\left(\cos\left(Z\right)*Y_{V}+\sin\left(Z\right)*X_{V}\right)$$

(4) $$X=X_{H}-\left(\sin\left(Z\right)*Y_{V}+\cos\left(Z\right)*X_{V}\right)$$

where X, Y and Z are the final measured Euler angles of the head and the indices H and V denote the corresponding devices attached to either the head or the vehicle.

#### 2.1.6. Software

**Figure 4:**
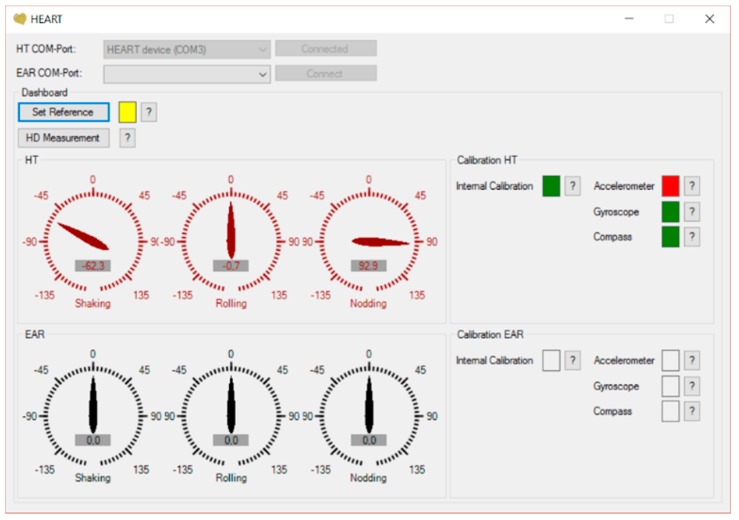
A snapshot of the software developed for the proposed head tracking system.

To assist with the calibration process, as well to display and export the data, in-house software was developed using Python (see Figure 4). Full calibration was carried out on both devices before the experiment began. The operator received real-time feedback regarding whether the calibration succeeded (green flag) or did not (red flag), for each axis, from the device's internal algorithms, as shown in the right part of Figure 4. This software only used a small proportion of the CPU resources to ensure that the camera-based head tracking system, where the video has High Definition (HD) quality, could work in parallel on a single laptop. To achieve this target, the software limited the sampling rate of both devices to 32 Hz. Although this is three times lower than the capability of the sensor, this is sufficient to capture all relevant head movements and to facilitate comparison with the data collected using the camera. 

### 2.2. Camera-Based Head Tracking

**Figure 5:**
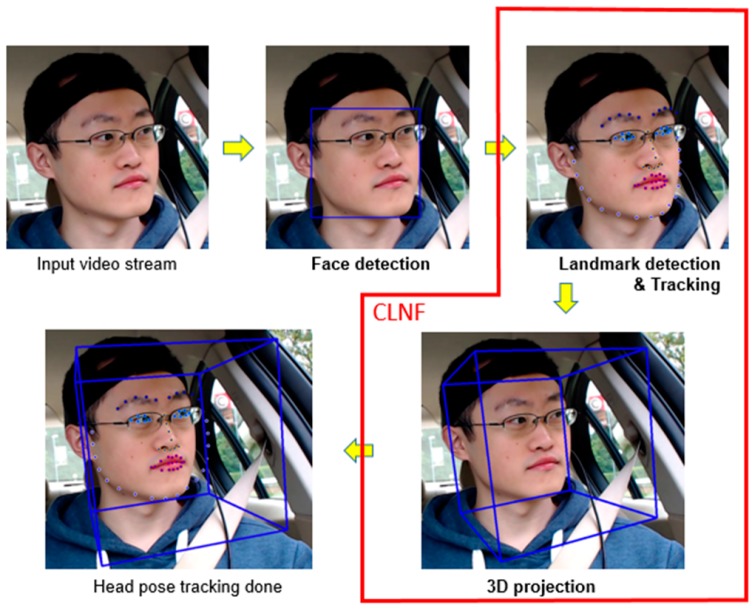
The process to measure head movement based on the camera-based head tracking system.

To validate and compare the results from the proposed system, this study also developed a camera-based head tracking system. The algorithm to analyse the data started with face detection to locate the face, followed by facial landmark detection and projecting 3D modelling. The overview of the algorithm is illustrated in Figure 5. For face detection, two state-of-the-art face detection methods were used: Dlib [24] and Pixel Intensity Comparisons Organised (PICO) [25]. Dlib defines a learning rule to score different locations and scales in an image by building feature pyramids. Through repeated smoothing and subsampling, a feature map is calculated for each pyramid level in each image. A coarse root filter is then used to roughly define an object and more delicate filters are used to define smaller parts of the object using a higher resolution. This results in a robust and accurate output for frontal face detection. PICO is an extension of the Viola-Jones object detection algorithm [26]. A joint of decision trees with pixel intensity comparisons is taken as binary tests for each binary classifier. A cascade of the classifiers will be slid through the whole image at every location and with all scales. If a region successfully matches every member of the cascade, it will be classified as a region of interest (ROI). The learning process consists of a greedy regression tree construction procedure and a boosting algorithm. This method does not require complex computation of integral images, image pyramid, Histogram of Oriented Gradients (HOG) pyramid or any other similar data structure. All binary tests in internal nodes of the trees are based on the same feature type. Having detected the ROI on the face, a recently developed method called Constrained Local Neural Field (CLNF) [27] was performed in Openface [28]. CLNF is an extension of Constrained Local Model (CLM), as proposed by Cristinacce and Cootes [29], that uses a probabilistic patch expert (landmark detector) to learn non-linear and spatial relationships between the input pixels and the probability of a landmark being aligned. Moreover, the fitting algorithm is replaced by the advanced non-uniform regularised landmark mean-shift method for optimising. This allows CLNF to deliver more reliable and efficient landmark detection. Having analysed the tracked facial landmarks, the variation pose and motion can easily be calculated [30]. As a result, a 3D model, including yaw, pitch and roll, can be built from these points of interest using a 3D projection, according to the camera parameters [28]. CLNF works on tracking by using a well-trained three-layer convolutional neural network (CNN) to predict landmark detection from previous frames in the video stream. If the CNN tracking fails, due to the observer leaving the view, the system will then reset to face detection.

## 3. Results

### 3.1. Accuracy Validation of a Single Device

**Figure 6:**
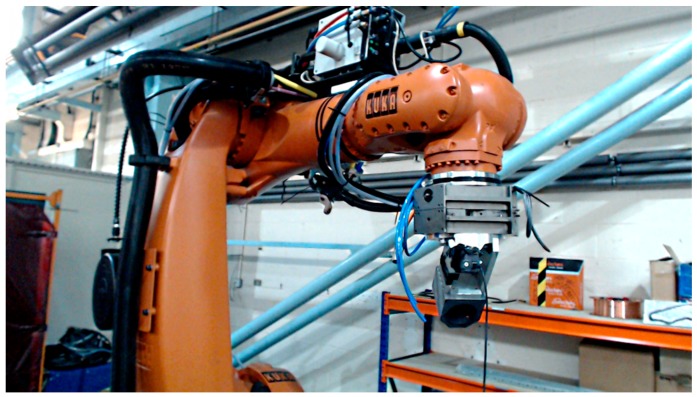
Test setup on the Kuka^®^ welding robot for accuracy validation of the proposed head tracking system.

To validate the accuracy of the device on a static platform, a test using a welding robot (Kuka^®^, Birmingham, UK) was carried out. The device developed in this project was firmly attached to the robot’s arm using adhesive tape, as illustrated in Figure 6. The main reason to employ this approach is that the robot can rotate precisely in any axis. The alignment of the device was ensured by rotating the device, while checking the software, until the orientation data read that the device was at the origin point. 

The robot was programmed to rotate to five different positions, with an offset of 25° between each one. The speed of rotation was chosen to be 30°/s, which is about four times slower than the average movement expected in non-driving tasks [31]. This should ensure that any dynamic effects were minimised during this test. All five positions were taken into consideration and the errors are averaged and are shown in Table 1. This shows that the average error for both *X* and *Z* axes were less than 0.4°, while the error for *Y* was considerably higher (about 1.5°). This may be due to the design of the device.

**Figure 7:**
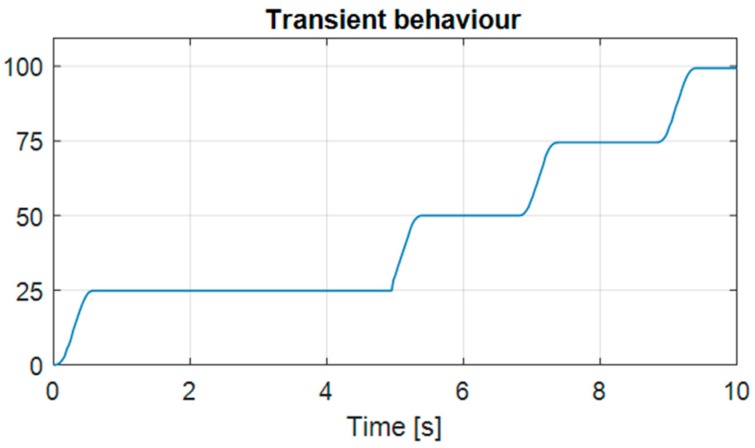
Transient behaviour of the head tracking system for step inputs of 25° each.

The transient behaviour of the tracking device can be described as over-dampened, meaning that the value was not overshooting. The measurement of *X* axis is shown in Figure 7 where no overshoot was observed, which suggests a good performance on transient behaviour. 

### 3.2. Calibration Error between Two Devices

When both devices were tested, there was always an error between the *Z* axes, because the magnetic sensor of each device were calibrated slightly differently. This means that as soon as the position, relative to the magnetic north pole, was changed a slight difference between the predicted positions of both devices would occur. To evaluate the effect of this behaviour, two fully calibrated devices were placed close to each other on a rigid mount. The short distance between the two devices ensured that the influence of local magnetic fields on the devices were as even as possible, so that the error would be cancelled out. The rigid mounting ensured that their relative positions were not changed during the test.

**Figure 8:**
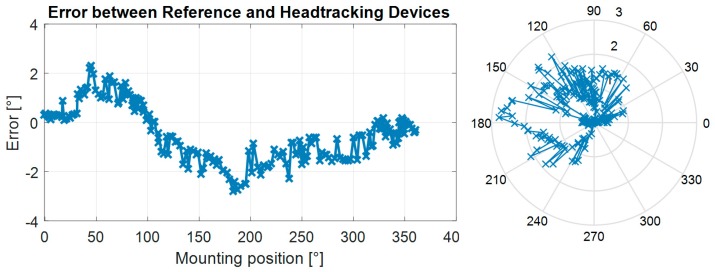
Calibration error between two devices in a linear (**left**) and circular form (**right**).

The test was carried out by turning the mount slowly more than 360° about the vertical axis, resulting in the collection of data for the shaking axis of both devices. The frame rate was 32 Hz and the test took about 5.2 s, which meant that each data point corresponds to a roughly 2° of change in the shaking axis. As shown in Figure 8 the biggest error occurred at the position opposite to the start position, and at that point was about 2.3°. This figure also shows that the error stayed close to zero for about 31.3°, because the sensor fusion algorithm can use gyroscopes to verify the exact movement for a certain period (in this case, 0.7 s). After this time, the gyroscope’s inaccuracies accumulate enough for the fusion algorithm to try to estimate the position using the magnetic sensors alone. This is when the error instantaneously increases to more than 1°. As shown in the right plot of Figure 8, the maximum error was smaller than 3°. It should also be noted that, rather than measuring the overall accuracy of the sensor, which has been conducted in Section 3.1, this section aimed to evaluate how another sensor would perform, relative to the tested device, and how they might affect each other in terms of electro-magnetic interference.

### 3.3. Indoor Testing

**Figure 9:**
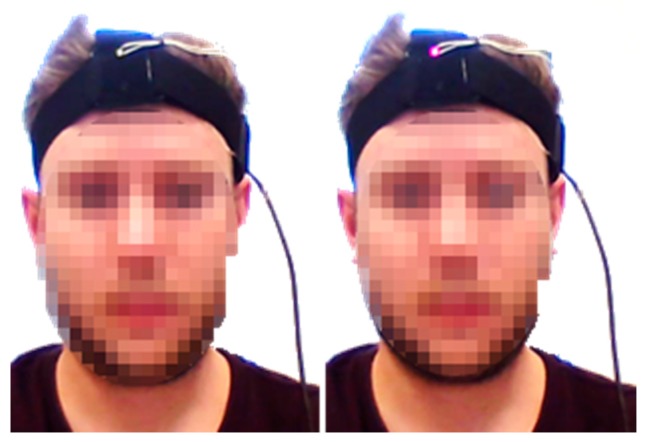
The LED Indicator used for synchronisation between two tracking systems. **Left**: Off, **Right**: On.

One large challenge associated with testing two head tracking systems, at the same time, is the synchronisation of the data acquisition process. To cope with this issue, an optical indicator, that was visible to the camera, was integrated into the head tracking system. One of the available output ports of the Uno R3 was used to power an LED at the front of the head that lit up as soon as recording of the new system began and switched off when the recording ended. Figure 9 shows two snapshots where the LED is on and off. 

**Figure 10:**
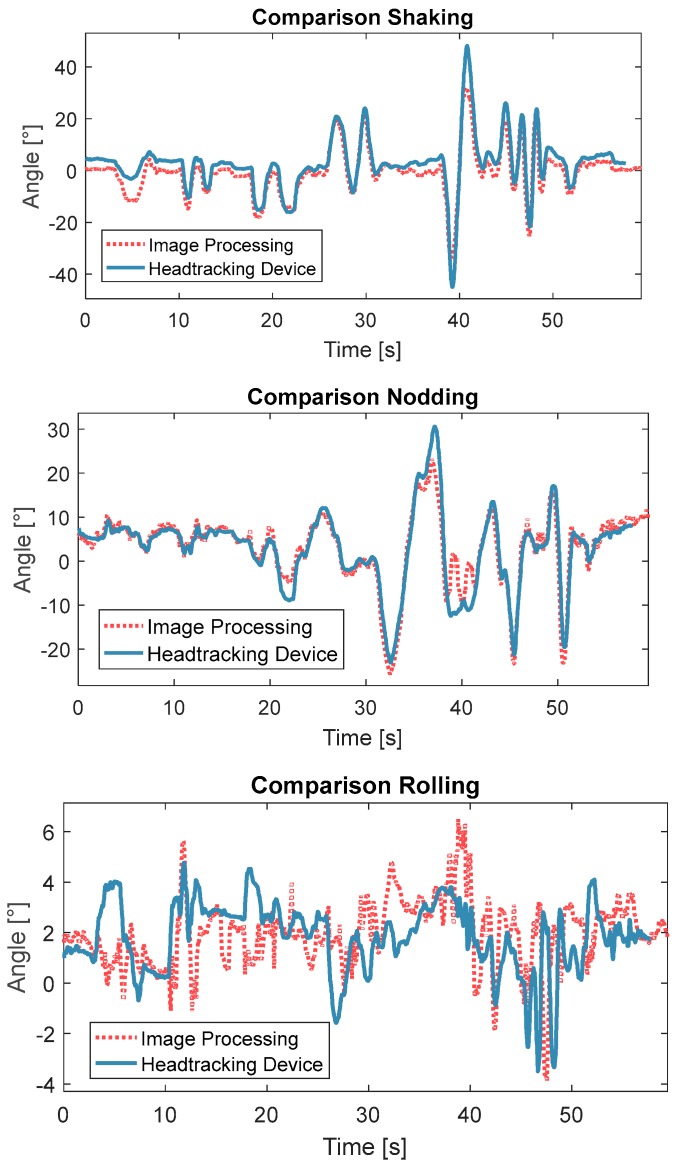
Comparison of head tracking for the proposed head tracking system (blue) and the camera-based tracking system (red) from the in-house experiments.

The experiments were conducted under the following conditions: (a) all movements in three axes were less than 45°; (b) no quick movements; (c) movements took place in the centre of the camera, and (d) no occlusion of the face. The experiments were conducted in a well-lit room in front of a window to partly simulate the conditions inside a vehicle. A Y50 Laptop (Lenovo^®^, Beijing, China) was used and its built-in camera used to record the images at 30 frames per seconds (fps) with a resolution of 1280 × 720 pixels. The results for the three axes for both head tracking systems are shown in Figure 10. The length of the data collection was 1 min. These results show that the measurements of shaking and nodding were highly similar between the two systems, while the measurement deviation for rolling was significant. This error was mainly caused by the developed sensor, which was more than four times higher than the error on the other two axes (see Table 1). Furthermore, the rolling movement in this experiment was very small, which results in the deviation between these two systems being more visually obvious. It has been observed from this experiment that the range of rolling ([−5°, 5°]) was significantly smaller than that for the other two axes ([−40°, 40°]). When the degree of shaking or nodding was larger than 20° or smaller than −20°, the camera-based system cannot measure movement accurately due to face detection failure. Clearly the camera-based system would have difficulties monitoring NDAs which included large movements. 

**Figure 11:**
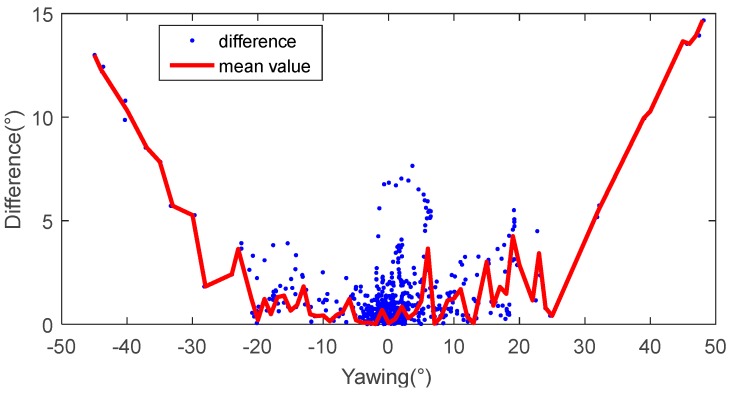
The absolute difference between the two tracking systems against different yawing angles from the in-house experiments.

To further analyse the performance, Figure 11 shows the differences between the two systems by angle in the clustering form (blue scatter), as well as by mean (red curve). This shows that if the shaking (yawing) angle is within the range of [−10°, −10°], the average difference is less than 2°, but increasing the angle increases the difference. Moreover, a small constant offset (2°–5°) between the two systems can be observed in the shaking measurement due to an error in the calibration of the two systems.

### 3.4. On-Road Testing

**Figure 12:**
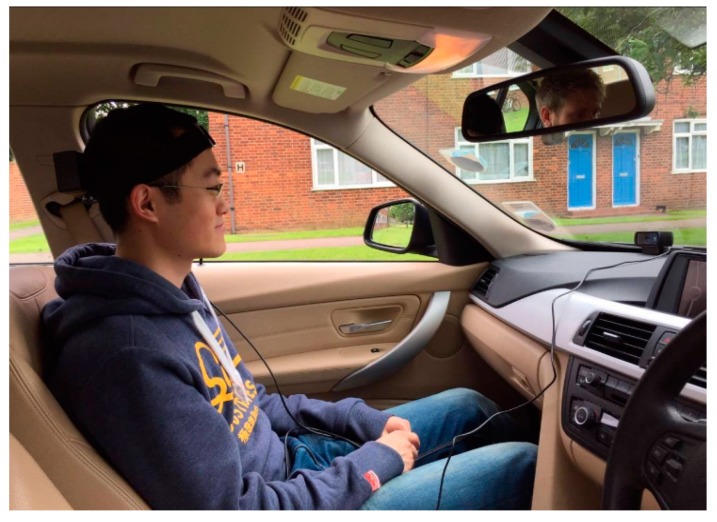
The experiment setup for the in-vehicle tests.

The proposed head tracking system and the camera-based tracking system were then trialled on-road. Figure 12 illustrates the experimental setup, where the person in the passenger seat was used to simulate the driver in a Level 3 autonomous vehicle. A C920 HD Pro camera (Logitech^®^, Newark, CA, USA) working under the resolution of 1280 × 720 pixels and the frame rate 30 fps, was fixed directly facing the face of the person being tested. The reference device for the head tracking system was located on the floor under the passenger seat and the second device was attached to the back of the person’s head. It should be noted that the LED indicator was facing the camera for synchronization, but was moved to the back during the testing phase, in order to avoid interference with the camera-based tracking system. All data were collected used a Lenovo^®^ Y50 Laptop with 2.2 GHz Intel-core i7 processor running on the Windows 10 operating system. The experiments were conducted under good illumination conditions to ensure adequate performance of the camera-based tracking system.

**Figure 13:**
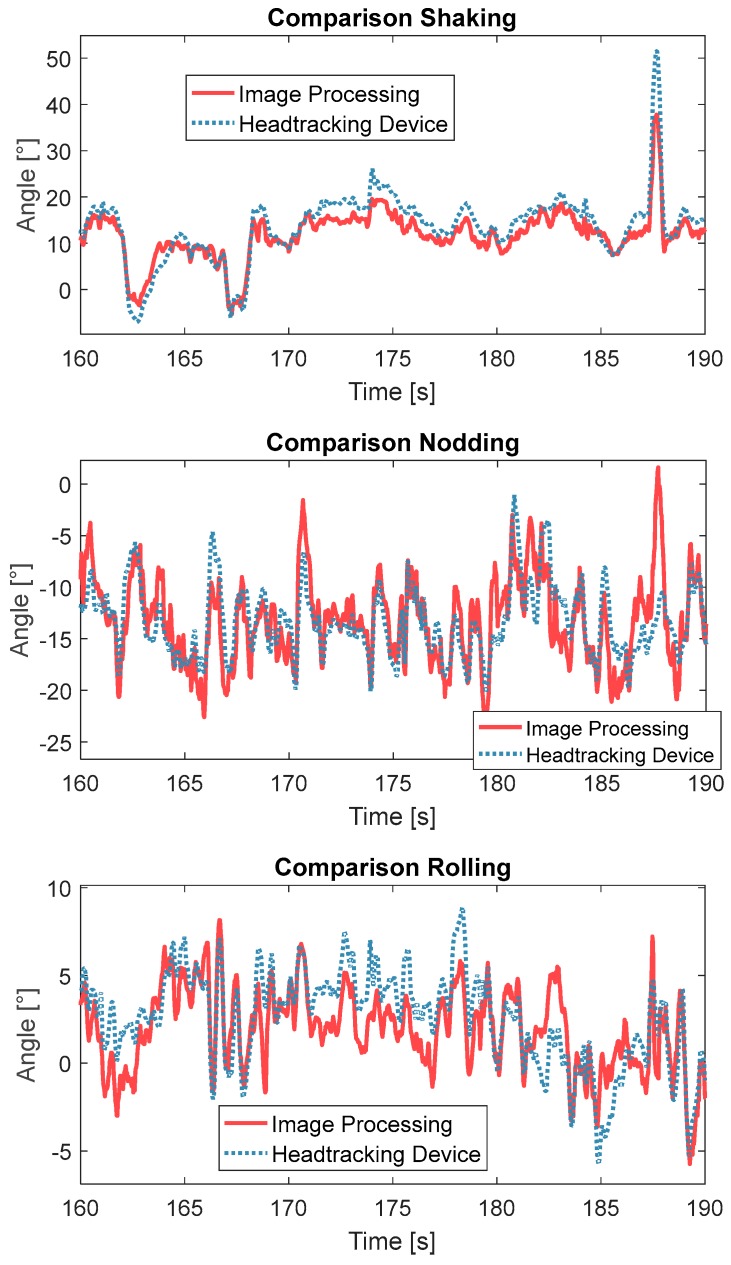
Comparison of head tracking for the proposed head tracking system (blue) and the camera-based tracking system (red) from the on-road experiments.

The two head tracking systems were compared across the three axes in an on-road test (see Figure 13). Similar results to those from the in-house test are seen here, in that the measurements of shaking and nodding were highly aligned while the measures of rolling were less well aligned. The rolling movement in this experiment was larger than found during the in-house test. Although the deviation of roll measurement was large, the observed patterns form the two systems were similar. When the degree of shaking was larger than 20°, the camera-based system was not able to accurately measure movement. In the last few seconds, on the nodding axis, the camera-based system lot track and jumped back to the origin, due to a large shaking movement (approximately 50°) where one-half of the facial features were not visible anymore. The drifts of shaking and rolling were more severe than those during the in-house test, due to the error from the reference device on the moving platform. Table 2 shows the averaged difference between the two tracking systems for the in-house and on-road tests. This shows that the average difference for the on-road tests were 2.45, 2.77 and 2.26 times higher than those found during the in-house test for shaking, rolling and nodding, respectively. According to a statistical test, the 95% confidence range was [1.85, 3.13].

### 3.5. A NDA Case Study

This section discusses the potential of the proposed head tracking system to characterise the driver behaviours using two NDAs. The two NDAs selected were “chatting with a passenger” and “using a smartphone”, which were tested inside a static vehicle. Three participants, comprised of two males and one female, were tested and each individual test was recorded for 5 min. The setup of the system was the same as that for the on-road test. 

**Figure 14:**
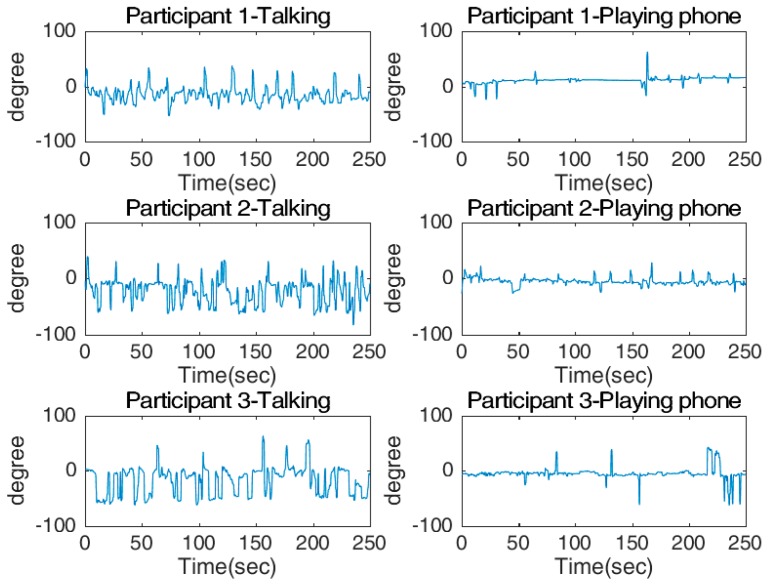
Measured shaking angles for two tasks: talking (**left** column) and playing phone (**right** column).

**Figure 15:**
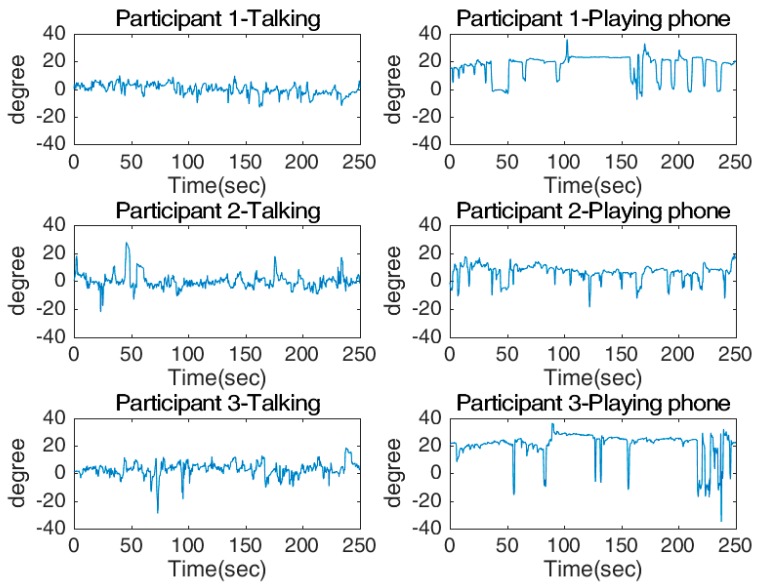
Measured nodding angles for two tasks: talking (**left** column) and playing phone (**right** column).

Figure 14 and Figure 15 show the shaking and nodding angles from the three participants. Figure 14 shows that for all three participants, the shaking angles during chatting regularly moved towards negative angles of up to 50°, while the shaking angles of playing with a phone were much more consistent and very close to zero. The opposite pattern was observed for the nodding angles, where the values were close to zero while chatting, but were mostly positive (about 20°) while playing with a phone. The histograms displaying the measured angles for all participants show that when they were “using a smartphone”, the shaking value had low variation and an average close to zero. In contrast, the shaking value for ‘chatting’ had a significant shift to one direction and the variation was larger. This observation was to be expected, as while chatting the participants regularly turned in the direction of the passenger. There were two obvious peaks in the shaking angle, one of which is about 0°, indicating that the participants were looking directly ahead, and the second which is at about 15°, indicating that the participants were facing towards the passenger. Furthermore, the mean for nodding was close to zero during chatting, while the mean while using a smartphone shifted significantly as the participants were constantly looking down at the phone.

**Figure 16:**
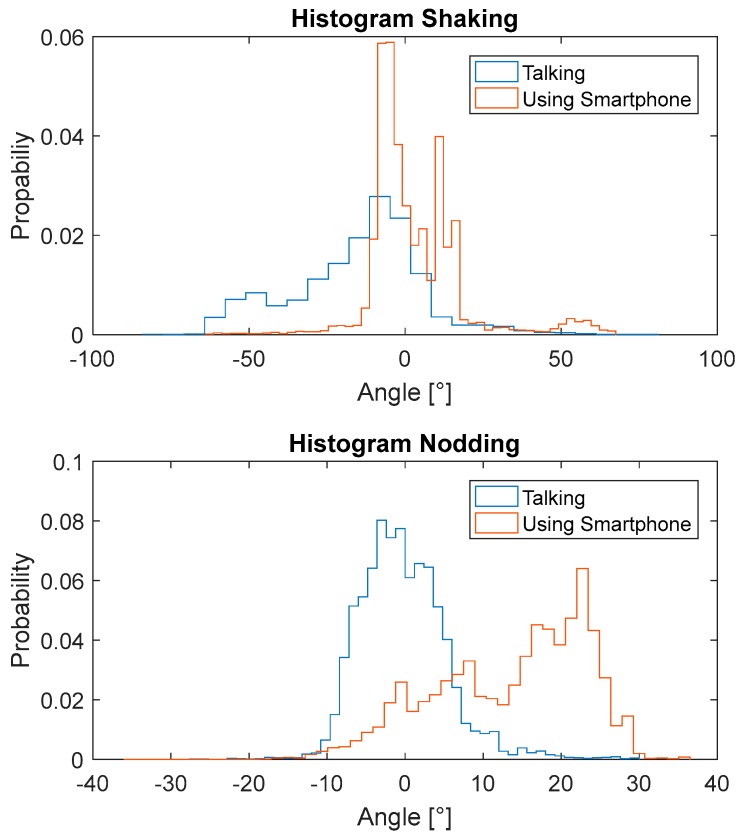
Histograms of the 3D angles for two tested NDAs.

In the previous section it was found that a single camera-based tracking system can only detect the shaking and nodding angles within about 20°. However, a much wider distribution of angles can be observed in Figure 16, which suggests an advantage for the system developed here. 

Table 3 shows the percentage of time when the angles were larger than 20°, which suggests that a single camera-based head tracking system could fail more than 40% of the time for the shaking angle during chatting and the nodding angle while using a mobile phone. 

## 4. Discussion and Conclusions

This paper proposed a novel head tracking system to monitor driver behaviour, particularly for drivers engaged in various non-driving tasks in a Level 3 autonomous vehicle. The whole system includes two identical integrated devices, attachment device and in-house software for system calibration and data collection. The main novelty of this system is the introduction of twin devices, one of which acts as the reference to measure the movement of vehicle, and the other which measures the absolute movement of the head. A LED indicator was employed to synchronise data capture between the camera-based tracking system and the system developed in this study. To validate this system, four experiments have been undertaken and produce the following conclusions:

- Through a test using a robotic arm, the averaged errors for the nodding, rolling and shaking axes of a single device on a static platform were 0.36°, 1.57° and 0.38° respectively.
- The in-house tests showed that the measures of shaking and nodding between the two systems were very close, with an average difference of less than 2°. However, when the angles were larger than 20°, the camera-based system could not measure the movement accurately, due to a face detection failure. These observations suggest that the system developed here would be more suitable, than the camera-based system, to measure head movements during NDAs which include large movements.
- The on-road test achieved similar results to the static in-car test. The only difference was that the average difference increased to 4.9°, 3.6° and 4.3° for the nodding, rolling and shaking axes respectively due to the error from the reference device.
- The case study in a static vehicle demonstrates the potential of the proposed system to characterise different NDAs based on head movement, particularly with regards to the shaking and nodding axes. The rolling axes was not used in this case study because: (a) the developed device had relatively low accuracy for rolling measurement, in comparison to shaking and yawing, and (b) it has been observed that the overall rolling value was much smaller than that for shaking and yawing, which leads to a reduced sensitivity to changes in NDA.
- The single-camera based head tacking system could misinterpret 40% of time when monitoring the selected NDAs, while the proposed head tracking system successfully overcame this problem.

It should be noted that this head tracking system was developed for ascertaining drivers’ head movements under different NDAs during research. The outcomes will be important for determining the required number and corresponding location of non-contact measures, such as camera and microphone, which are more suitable for real applications to identify NDAs. The developed tracking system can be enhanced by improving the accuracy of the rolling measurement and conducting the calibration process automatically.

## Figures and Tables

**Table 1:** Accuracy validation for the proposed device.

	X (°)	Y (°)	Z (°)
**Average Error**	0.36	1.57	0.38
**Standard Deviation**	0.13	0.68	0.23
**Maximal Error**	0.55	2.09	0.63

**Table 2:** Averaged difference of the two tracking systems between the in-house and on-road tests.

Axis	On-Road Tests (°)	In-House Tests (°)
Shaking	4.9	2.0
Rolling	3.6	1.3
Nodding	4.3	1.9

**Table 3:** The percentage of time when the angles were larger than 20°.

Axis	Chatting	Playing Phone
Shaking	44.61%	0.93%
Nodding	4.74%	42.57%
